# High Expression of Acid-Sensing Ion Channel 2 (ASIC2) in Bone Cells in Osteoporotic Vertebral Fractures

**DOI:** 10.1155/2019/4714279

**Published:** 2019-08-19

**Authors:** Ching-Yu Lee, Tsung-Jen Huang, Meng-Huang Wu, Yen-Yao Li, Kuan-Der Lee

**Affiliations:** ^1^Department of Orthopedics, Taipei Medical University Hospital, Taipei, Taiwan; ^2^Department of Orthopaedics, School of Medicine, College of Medicine, Taipei Medical University, Taipei, Taiwan; ^3^Graduate Institute of Clinical Medical Sciences, College of Medicine, Chang Gung University, Taoyuan, Taiwan; ^4^Department of Orthopedic Surgery, Chang Gung Memorial Hospital, Chiayi, Taiwan; ^5^Division of Hematology and Oncology, Department of Internal Medicine, Taipei Medical University Hospital, Taipei, Taiwan; ^6^Department of Internal Medicine, School of Medicine, College of Medicine, Taipei Medical University, Taipei, Taiwan

## Abstract

Little is known about the function of acid-sensing ion channels (ASICs) in bone cells or osteoporotic vertebral fractures (OVF). This study delineated ASICs expression in adult human bone marrow-mesenchymal stem cells- (BM-MSC-) derived osteoblasts and in OVF bone cells. Adult BM-MSC-derived osteoblasts were isolated and cultured in different pH values. Osteogenic markers as alkaline phosphatase (ALP), osteopontin (OPN), and osteocalcin (OC) mRNA were assessed. Western blots method was applied to analyze ASICs protein expression in different pH values. Amiloride was added into the osteogenic media to analyze the Na^+^/K^+^ ATPase change. We harvested the vertebral cancellous bone through a bone biopsy needle in 26 OVF patients when performing percutaneous vertebroplasty. Six vertebral bone specimens obtained from 4 patients with high-energy vertebral fractures were used as the control. The reverse transcription polymerase chain reaction was performed to analyze the quantitative mRNA expression of ASICs. Osteogenic markers as ALP, OPN, and OC mRNA were higher expressed in increasing pH values throughout osteoblastogenesis. ASIC proteins were higher expressed in lower pH media, especially ASIC3, and ASIC4. The highest protein expression at days 7, 14, and 21 was ASIC2, ASIC4, and ASIC3, respectively. Expression of Na^+^/K^+^ ATPase was significantly decreased in cultured osteoblasts by addition of amiloride into the pH 6.9 osteogenic media. ASIC2 mRNA was most highly expressed with a 65.93-fold increase in the biopsied vertebral bone cells in OVF compared with the control. In conclusion, we found osteoblastogenesis was reduced in an acidic environment, and ASIC2, ASIC3, and ASIC4 were most highly expressed in turn during osteoblastogenesis within acidic media. ASIC2 was the most abundantly expressed gene in human bone cells in OVF compared with the control. ASIC2 could be crucial in the pathogenesis of osteoporosis and could serve as a therapeutic target for antiosteoporotic therapies.

## 1. Introduction

Osteoporosis is an age-related skeletal disease characterized by decreased bone mass and deteriorated microarchitecture of the bone tissue that contribute to an increased risk of fragile fractures [1, 2]. The most common osteoporotic fractures are vertebral, hip, and wrist fractures that result in morbidity and mortality in the elderly [3]. Biomechanism of osteoporosis is homeostatic imbalance in osteoclast-mediated resorption and osteoblast-mediated formation of the bone. The secretion of protons by osteoclasts during bone resorption leads to acidification of the osteoclast-bone interface [4]. In addition, the activities of bone cells can be regulated by changes in pH. An increasing pH can induce mineralization and osteoblastic activity [5]. Acidosis, by contrast, stimulates osteoclastic bone resorption [6]. An acidic microenvironment can induce bone loss by increasing osteoclastogenesis [7, 8], inducing autophagy in osteoblasts [9], and inhibiting osteoblast-mediated biomineralization [10]. With increasing age, a significant increase in the steady-state blood hydrogen ion concentration and reduction in steady-state plasma bicarbonate concentration were observed, indicating a progressively worsening low-level metabolic acidosis [11]. Hence, extracellular acidosis may play an important role in osteoporosis development.

Acid-sensing ion channels (ASICs) are a subfamily of epithelial sodium channel/degenerin and are recognized as tissue pH sensors [12, 13]. ASICs are transcripted and translated from 5 genes that encode 7 subunits of ASIC noted (ASIC1a, ASIC1b, ASIC2a, ASIC2b, ASIC3, ASIC4, and ASIC5). Most of ASICs were activated by rapidly increasing extracellular concentration of hydrogen ion (acidic pH) while ASIC5 appears to be sensitive to bile acids rather than protons [14]. In 1997, Waldmann et al. identified the ASIC as a proton-gated cation channel involved in acid sensing and found that the ASIC is expressed in the dorsal root ganglia and is distributed widely throughout the brain [15]. Multiple studies have focused on the relevance between ASICs and the pathophysiology of nonneuronal tissue diseases, such as pulmonary cystic fibrosis [16], inflammatory bowel disease [17], liver fibrogenesis [18], immunobiology of dendritic cells [19], arthritis [20, 21], and intervertebral disc degeneration [22]. However, little is known about whether the expression of ASICs is relevant to the pathophysiology of osteoporosis. This study aimed at delineating the expression pattern of ASICs in adult human bone marrow-mesenchymal stem cells- (BM-MSC-) derived osteoblasts and the expression pattern of ASICs in human bone cells in osteoporotic vertebral fractures (OVF).

## 2. Materials and Methods

### 2.1. Human Bone Marrow MSC Isolation and Culture

To isolate human MSCs, bone marrow aspirates were taken from the iliac crest of normal adult donors after informed consent and under a protocol approved by an Institutional Review Board (IRB/CGMH reference no. 97-0230B).

The techniques for isolation and culture of the adult bone marrow MSCs were previously described by our colleague, Lee KD [23]. MSCs are cultured and maintained in expansion medium consists of Iscove's modified Dulbecco's medium (IMDM, Gibco BRL, Grand Island, NY) with 10% fetal bovine serum (Hyclone, Logan, UT) supplemented with 10 ng/mL epidermal growth factor (R&D Systems), 10 ng/mL beta fibroblast growth factor (R&D Systems), and 1% penicillin- streptomycin- glutamine (Gibco BRL). Cells are allowed to adhere overnight, and nonadherent cells are washed out with medium changes. Medium changes were performed twice weekly thereafter. Cultures were incubated at 37°C in a humidified atmosphere of 5% CO_2_ and 95% air until reaching cell confluence.

### 2.2. Osteoblast Differentiation

To induce osteogenic differentiation, the MSCs cells were treated with osteogenic medium in different pH levels (pH of 6.9, 7.4, and 8.0) for three weeks with medium changes twice weekly. Osteogenic medium consists of Dulbecco's modified Eagle's medium (DMEM, Gibco BRL, Grand Island, NY) supplemented with 0.1 nM dexamethasone (Sigma-Aldrich, St. Louis, MO), 10 mM *β*-glycerophosphate (Sigma-Aldrich, St. Louis, MO), and 50 *μ*M L-ascorbic acid 2-phosphate (Sigma-Aldrich, St. Louis, MO). Osteoblastogenesis was evaluated by colorimetric semiquantitative assessment of alkaline phosphatase activity as well as Alizarin Red S staining. In addition, amiloride (Sigma-Aldrich, St. Louis, MO), an ASICs antagonist, the ASIC channel can be blocked by amiloride. An 0.2mM amiloride will be added into osteogenic culture median with different pH (pH 6.9, 7.4), in which the MSCs are incubated for 3, 6, and 12 hours to analyze the differences of Na^+^/K^+^ ATPase change.

### 2.3. Osteoporotic and Nonosteoporotic Bone Tissue Collection and Processing

Osteoporosis was defined as bone marrow density (BMD) at the hip or lumbar spine less than or equal to 2.5 standard deviations (SDs) below the mean BMD of a young adult reference population or a fragility fracture of a vertebra or hip and BMD between 1.0 and 2.0 SDs below the mean BMD of a young adult reference population [24]. Bone density was measured through dual energy X-ray absorptiometry. A bone biopsy needle (Stryker 11G Match Ground Bevel Tip Introduction Needle 5′′, Stryker Corporation, Michigan, USA) was inserted into the vertebral body through the transpedicular route to obtain the vertebral cancellous bone tissue. The biopsied cancellous bone specimens from OVF were collected during spine surgery of 26 patients (22 females and 4 males, with a mean age of 78 years) with low-energy trauma. Of the 26 patients with fragile vertebral fractures, the T-score of −2.5 SDs or lower was noted in 23 patients and the T-score between −1 and −2.5 SDs was noted in 3 patients. The six biopsied cancellous bone specimens at nonfracture vertebral levels from 4 patients (2 females and 2 males, with a mean age of 45 years), who underwent surgery for high-energy spine fractures, were used as the control. The 4 patients in the control group had a T-score of −1 SD or above, which was considered as normal bone density. The biopsied cancellous bone specimen was flushed with saline to remove blood clots and then was immediately frozen in liquid nitrogen following surgery and stored at −80°C until further use. Approval for this study was obtained from the ethics committee and institutional review board of the institution (CMRP IRB no. 101-0135C).

### 2.4. RNA Extraction and Reverse Transcription Polymerase Chain Reaction (RT-PCR)

Total cellular RNA was extracted from the mesenchymal stem cells after differentiation at days 7, 14, and 21 or the biopsied cancellous bone cells using the illustra RNAspin Mini RNA Isolation Kit (GE Healthcare) according to the manufacturer's instructions. The mRNA expression was detected using RT-PCR and quantitative RT-PCR (qRT-PCR).

RevertAid™ First Strand cDNA Synthesis Kit (Fermentas, Life Science) was used in reverse transcription (RT) reaction according to the manufacturer's instructions. Amplification with specific primers was performed in a GeneAmp PCR system 9600 (Perkin Elmer) for 35 cycles with a 15 seconds/94°C denaturation, 30 seconds/Primer Tm-5°C annealing, 1 minute/72°C extension profile in the case of glyceraldehyde-3-phosphate dehydrogenase (GAPDH), alkaline phosphatase (ALP), osteopontin (OPN), osteocalcin (OC), and Na+/K+ ATPase. The oligonucleotide primers for PCR were based on published sequences of mRNAs (Table 1). Amplified products were electrophoresed on 2% agarose gel stained with ethidium bromide. The band intensity of the PCR products was quantified using the Image-Pro Plus, normalized to the density of GAPDH band.

**Figure 1:**
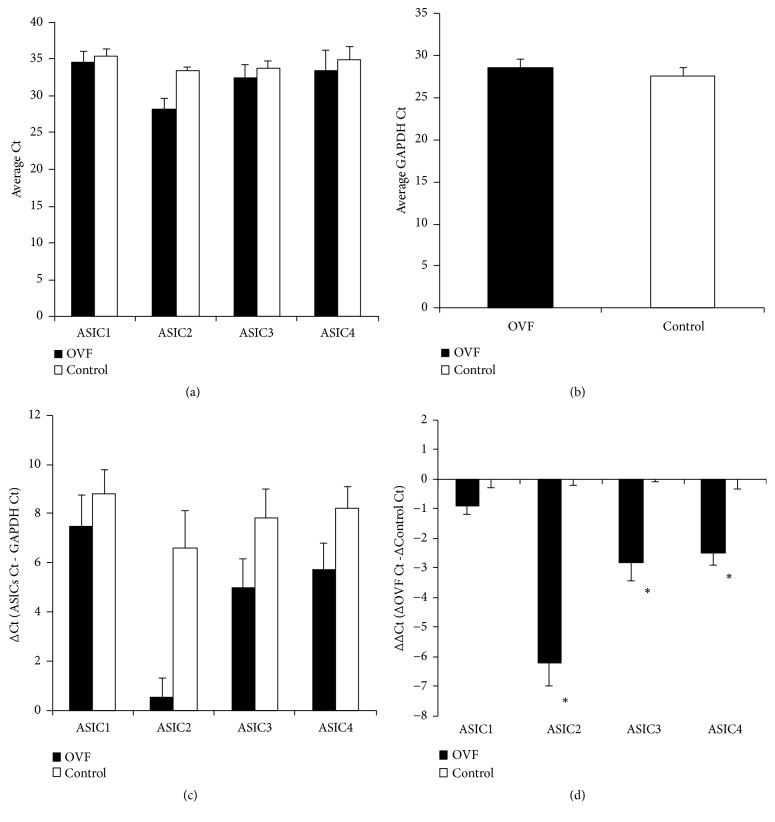
*Threshold cycle (Ct) of 4 subtypes of acid-sensing ion channels (ASICs, ASICs 1, 2, 3, and 4) and glyceraldehyde-3-phosphate dehydrogenase (GAPDH) determined using the qRT-PCR from human cancellous bone cells in osteoporotic vertebral fractures (OVF) and nonosteoporotic bone (the control)*. (a) The average C*t *of ASICs 1, 2, 3, and 4. (b) The average C*t *of GAPDH. (c) ΔC*t *values of the 4 subtypes of ASICs differentially expressed in cancellous bone cells between OVF and the control. (d) The fold change in ASIC mRNA between OVF and the control was expressed using the 2^−ΔΔCt^ method. *∗*p<0.05.

The qRT-PCR was performed using double-stranded DNA-binding fluorescence dye SYBR Green I and the iCycler iQ Detection System (Bio-Rad Laboratories, Hercules, CA). Primers for the qRT-PCR on a computer (Beacon Designer 2.1; Premier Biosoft Int., Palo, CA) were designed and synthesized by Mission Biotech Co., Ltd. (Taipei, Taiwan) (Table 1). The relative gene expression was determined by the 2^−ΔΔCt^ method and human GAPDH was used as an internal control gene. The qRT-PCR revealed a value (Ct) denoting the threshold cycle of specific target gene amplification at which the PCR product was first detected through fluorescence (Figure 1). All the experiments were performed in duplicate or triplicate.

### 2.5. Antibodies

Primary antibodies against ASIC subunits were purchased from Millipore (Chemicon, Millipore Corporation, USA) and used in 1:500 (ASIC1, ASIC4), 1:200 (ASIC2), 1:800 (ASIC3), and 1:2000 (GAPDH) dilutions for Western blotting.

### 2.6. Protein Extraction and Western Blotting

The harvested cells (the mesenchymal stem cells after differentiation at days 7, 14, and 21) were washed three times in ice-cold PBS and centrifuged at 1200 rpm for 5 min at 4°C. Cell pellets were resuspended in mammalian protein extraction reagent (M-PER, Thermo Scientific, IL, USA) with protease inhibitors for 30 min on ice and then centrifuged at 14000 rpm for 15 min. The supernatant was collected and total protein was determined using the BCA (bicinchoninic acid) Protein Assay Kit (Pierce, IL, USA). Next, 50*μ*g total protein extract was electrophoresed in 10% SDS-polyacrylamide gel electrophoresis for ASICs and electrotransferred onto polyvinylidene difluoride (PVDF) membranes. After blotting, membranes were blocked with 1X TBS/Casein Blocker (Bio-Rad, Hercules, CA) for 1 hour at room temperature and incubated overnight at 4°C with primary antibody. Primary antibodies were allowed to equilibrate for 2 h then the blots were washed 3 times, 10 min each, with TBS, pH 7.4 containing 0.05% Tween 20. Secondary antibodies were either goat anti-mouse or goat anti-rabbit HRP conjugated monoclonal antibodies (Chemicon, Millipore Corporation, USA) and were used at a dilution of 1:50,000. Proteins were visualized on imaging film (Biomax, Eastman Kodak Company, Rochester, NY) by chemiluminescence using ECL Western Blotting Analysis System (Chemicon) and densitometry scanning were performed using Image-Pro Plus 4.5 software (Media Cybernetics, Silver Spring, USA).

### 2.7. Statistical Analysis

Statistical analyses were performed using the Statistical Package for the Social Sciences for Windows (SPSS, version 12.0). The Wilcoxon rank sum test was adopted for every continuous variable. A repeated measurement of analysis of variance was performed to examine the difference in expression of ASIC mRNA between the two groups. All tests were two sided, and the significance level was set at* P* < 0.05 for each test.

## 3. Results

### 3.1. Bone Marrow-Derived MSCs Differentiate into Mature Osteoblasts

**Figure 2:**
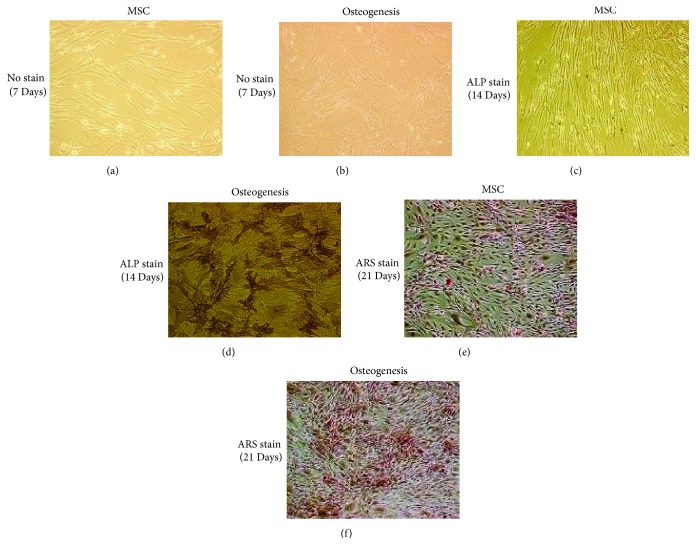
*Bone marrow-derived MSCs differentiate into mature osteoblasts*. The adult human bone marrow-derived mesenchymal stem cells (MSCs) were cultured in the osteogenic medium. Osteoblastogenesis is assessed by alkaline phosphatase (ALP) and Alizarin red S (ARS) staining. (d) and (f) Under the induction of osteogenic medium for 21 days, bone marrow-derived MSCs could differentiate into osteoblasts showing positive alkaline phosphatase and Alizarin red S staining.

Osteogenesis is assessed by Alkaline Phosphatase (ALP) and Alizarin red S (ARS) staining (Figure 2). Under the induction of osteogenic medium for 21 days, BM-derived MSCs could differentiate into osteoblasts showing positive alkaline phosphatase (Figure 2(d)) and Alizarin Red S staining (Figure 2(f)).

### 3.2. Osteoblastogenesis Is Reduced in an Acidic Environment

**Figure 3:**
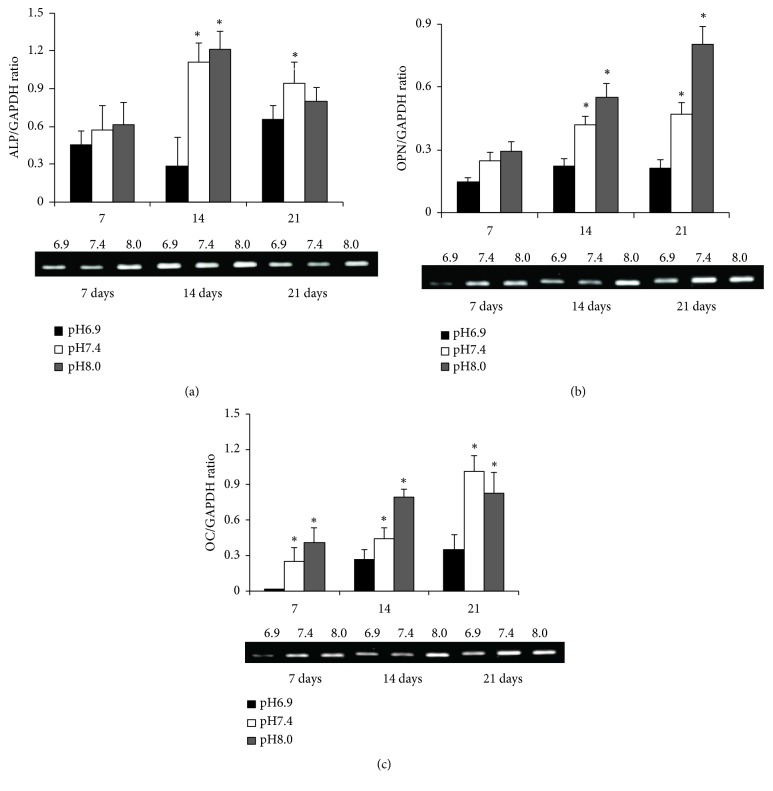
*Inhibition of osteoblastogenesis in an acidic environment*. Osteoblast differentiation of mesenchymal stem cells (MSC) was treated with osteogenic media in different pH levels (pH of 6.9, 7.4, and 8.0). Detection of osteogenic markers, including alkaline phosphatase (ALP), osteopontin (OPN), and osteocalcin (OC), in MSC-derived osteoblasts after differentiation (days 7, 14, and 21) were detected by reverse transcription polymerase chain reaction. (a), (b), and (c) The pH-dependent osteoblast markers as ALP, OPN, and OC were observed with a higher expression at increasing pH values throughout osteoblast differentiation. *∗*p<0.05.

A pH-dependent osteogenic markers as alkaline phosphatase (ALP), osteopontin (OPN), and osteocalcin (OC) were observed with a higher expression at increasing pH values throughout osteoblast differentiation (Figure 3).

### 3.3. Expression of ASICs in Osteoblasts Is Induced in an Acidic Environment

**Figure 4:**
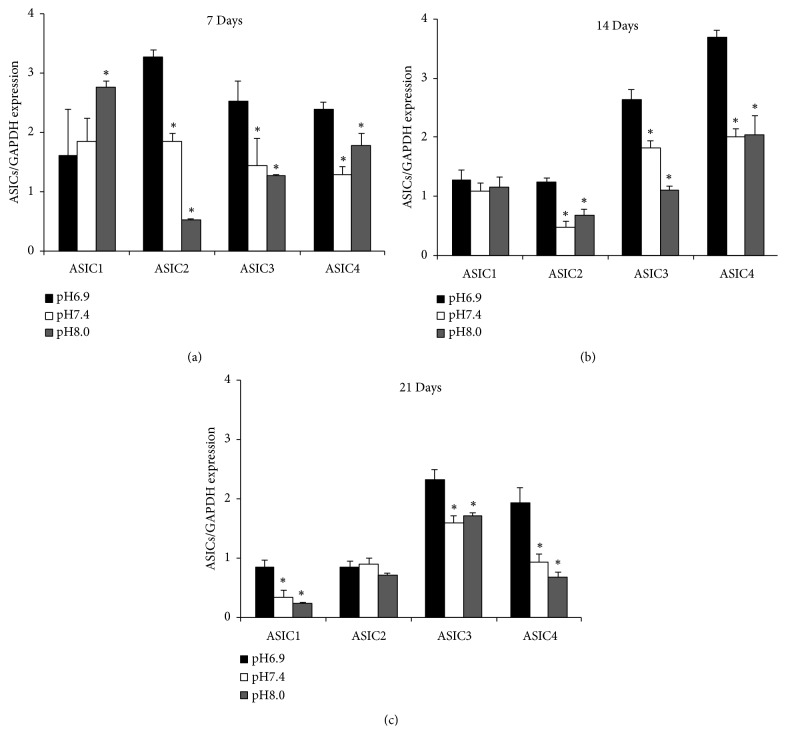
*Promotion of expression of ASICs in osteoblasts in an acidic environment*. Western blots were performed to analyze ASIC1, ASIC2, ASIC3, and ASIC4 expression of osteoblasts in pH-dependent media. ASIC1, ASIC2, ASIC3, and ASIC4 were all expressed in osteoblasts. Higher expression of ASIC proteins during osteoblast differentiation in lower pH (pH 6.9) medium condition, especially more abundant levels in ASIC3 and ASIC4 expression. The most highly expressed protein at days 7, 14 and 21 was ASIC2, ASIC4, and ASIC3, respectively. *∗*p<0.05.

ASIC1, ASIC2, ASIC3, and ASIC4 were all expressed in osteoblasts. Higher expression levels of these proteins were found in lower pH medium condition, especially in ASICs 3 and 4 by Western blotting method. The highest protein expressions at days 7, 14, and 21 were ASIC2, ASIC4, and ASIC3, respectively (Figure 4).

### 3.4. ASICs Activities Are Inhibited by Amiloride in an Acidic Environment

**Figure 5:**
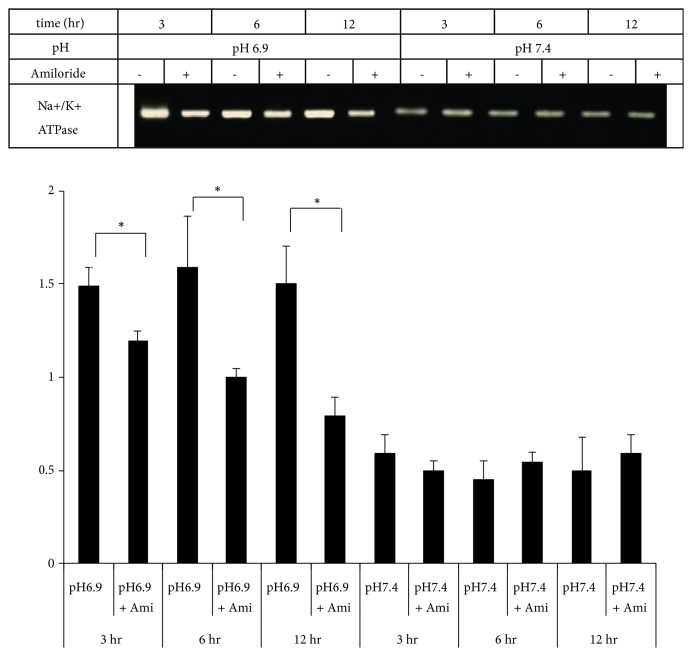
*Inhibition of ASICs activities by amiloride in an acidic environment*. A 0.2mM amiloride (ASIC antagonist) was added into the osteogenic medium culture (pH of 6.9, 7.4, 8.0) on the 7 days and incubation 3hr, 6hr, and 12hr to analyze the differences of Na^+^/K^+^ ATPase change. Normally, Na^+^/K^+^ ATPase RNA content was significantly increased in the pH 6.9 cultured osteoblasts. While amiloride was added into the osteogenic medium, it caused a significant decrease in Na^+^/K^+^ ATPase in the pH 6.9 cultured osteoblasts. *∗*p<0.05.

In studies of neural nociceptors, it has been noted that ASICs can be blocked by a diuretic agent,** a**miloride, and** a**miloride can be served as an antagonist of ASICs by blocking Na^+^/K^+^ channel [25–27]. Normally, Na^+^/K^+^ ATPase RNA content was significantly increased in the pH 6.9 cultured osteoblasts. While amiloride was added into the osteogenic medium, it caused a significant decrease in Na^+^/K^+^ ATPase in the pH 6.9 cultured osteoblasts (Figure 5).

### 3.5. Expression of ASIC2 Is Predominant in Osteoporotic Bone Cells

**Figure 6:**
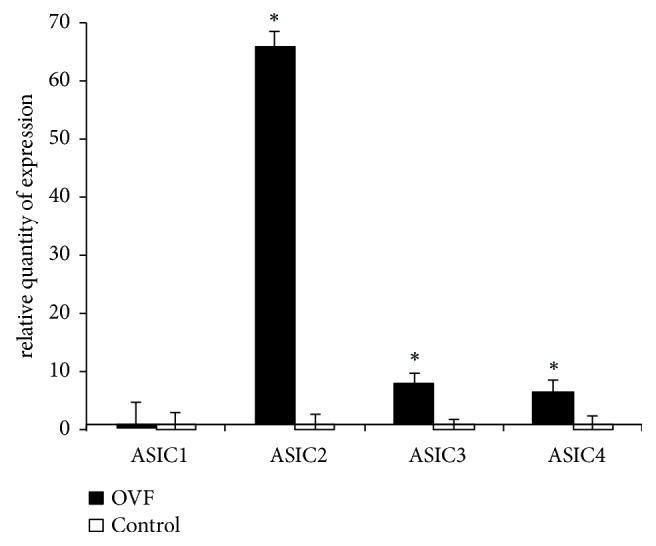
*The fold increase of each subtype of ASICs (ASICs, ASICs 1, 2, 3, and 4) expression, using the *2^−ΔΔ*Ct*^* method, from human cancellous bone cells in osteoporotic vertebral fractures (OVF) and nonosteoporotic bone (the control)*. The ASIC2 was the gene with the highest expression, with a 65.93-fold increase in the biopsied cancellous bone cells in OVF compared with the control. ASICs 1, 3, and 4 were expressed with 0.35-, 7.90-, and 6.46-fold increases, respectively. *∗*p<0.05.

ASIC2 was the gene with the highest expression, with a 65.93-fold increase in the biopsied cancellous bone cells in OVF compared with the control. ASICs 1, 3, and 4 were expressed with 0.35-, 7.90-, and 6.46-fold increases, respectively (Figure 6).

## 4. Discussion

In the current study, pH-dependent osteogenic markers as alkaline phosphatase (ALP), osteopontin (OPN), and osteocalcin (OC) were observed with a higher expression at increasing pH values throughout osteoblast differentiation. There was no study in the literature to investigate pH-dependent expression of ASICs in osteoblastogenesis. We found higher expression of ASIC proteins during osteoblast differentiation in lower pH (pH 6.9) medium condition, especially more abundant levels in ASIC3 and ASIC4 expression. We also found that the most highly expressed proteins at days 7, 14, and 21 were ASIC2, ASIC4, and ASIC3, respectively. In 2005, Jahr and colleagues [5] were credited for the first time to demonstrate ASICs in human skeletal cells. Similar to our findings, the onset of mineralization was earlier and reached higher level as the extracellular pH level was increasing. By applying the qRT-PCR, Jahr et al. used human osteoarthritic femoral head biopsy specimens and found that expressions of ASIC2 and ASIC3 in bone cells were the highest while in chondrocytes it was ASIC1. Furthermore, in the current study, we found that Na^+^/K^+^ ATPase mRNA content was significantly increased in low pH cultured osteoblasts. While amiloride was added into the osteogenic media, it caused a significant decrease in Na^+^/K^+^ ATPase in the pH 6.9 cultured osteoblasts. Thus, bone homeostasis is regulated through ASICs in the bone as bone cells can sense changes in extracellular pH because of ASICs. Osteoblastogenesis would be reduced in acidic environment via the expression of ASIC2, ASIC3, or ASIC4 in osteoblasts.

A paucity of data exists regarding whether the type of ASICs in human osteoporotic bone marrow cells is different from that in human nonosteoporotic bone marrow cells. Kanaya and colleagues [28] found that the expression levels of ASICs 1 and 2 were significantly increased in the bone marrow stromal cells of ovariectomized mice compared with sham mice. Our study was the first to analyze ASICs in human bone cells in OVF, and ASIC2 was the gene with the highest expression, with a 65.93-fold increase in the bone cells in OVF compared with the control. Because bone marrow cells consist of various cell types, ASIC expression in bone marrow biopsies could not directly refer to a distinct cell type. Jahr and colleagues [5] used human peripheral blood mononuclear cells treated with macrophage colony-stimulating factor and receptor activator of the NF-*κ*B ligand as human osteoclasts and found that ASIC2 was the most abundantly expressed gene in differentiated osteoclasts. By contrast, ASIC2 expression was significantly lower during osteoblast differentiation and mineralization. Therefore, the clinical relevance of the findings from these in vivo studies indicates that ASIC2 may be crucial in osteoclast bone resorption in the pathophysiology of osteoporosis. Moreover, in this study, in vitro human osteoblast culture showed that ASIC3 and ASIC4 were abundantly expressed in a low pH level and osteoblastogenesis was reduced in an acidic medium, and in vivo analysis of vertebral bone cells showed that expression of ASIC3 and ASIC4 was also increased in OVF cells compared to non-OVF bone cells. These results indicated that ASIC3 and ASIC4 still play a role in decreased osteoblastogenesis in an acidic environment such as osteoporosis. Expression of ASIC1 fluctuated during osteoblastogenesis in an acidic environment and in the osteoporotic bone cells compared to the control. The role of ASIC1 may remain unclear in bone.

To date, little is known about the function of ASICs in bone cells or OVF. ASIC2, which consists of two splice variants, ASIC2a and ASIC2b, is widely expressed in both central and peripheral nervous systems, such as the amygdala, hippocampus, brain stem, spinal cord, and dorsal root ganglion [11, 29, 30]. In addition, ASIC2 is implicated in peripheral mechanosensitive sensory neurons and mechanoreceptors, including aortic baroreceptor [31], pressure-induced vasoconstriction in cerebral vessels [32], and Pacinian corpuscles [33]. Unlike the numerous reports on the role of ASIC2 in neuron tissues, the presence and function of ASIC2 in the skeletal system were still unclear. ASIC2 was the most frequently expressed in human intervertebral disc cells compared with other ASICs and participated in disc degeneration [34]. In addition to extracellular acidity, mechanical loading can influence bone homeostasis. Mechanical stress sensed by osteocytes is converted into biochemical signals to regulate osteoclast and osteoblast activities [35]. Pathological overload occurs in an osteoporotic status when microcracks propagate in the cancellous bone during compression overloading [36]. ASIC2 serves as a mechanoprotein in certain cell types [31–34], and we have observed in this study that ASIC2 was significantly highly expressed in osteoporotic bone cells than in nonosteoporotic bone cells. The molecular and genetic regulation of mechanosensitivity is extremely complex, and further studies are required to investigate the correlation between ASIC2 and mechanotransduction in bone homeostasis.

There were some limitations in this study. First, the cancellous bone tissue from the vertebrae constitutes various cell types, including osteoblasts, osteoclasts, and hematopoietic cells, which may present a challenge in data interpretation. To eliminate the interference factors, the cancellous bone harvested from the vertebrae was flushed to remove blood elements before freezing it. Second, obtaining bone biopsies from a healthy human is difficult. To obtain healthy bone tissues from patients with acute high-energy spine trauma, a bone biopsy at the nonfracture vertebrae was performed during pedicle screws placing surgeries and all with patients' informed consent. Third, the age of OVF patients was higher than that of non-OVF patients. There was difficulty in matching age comparability. Osteoporotic vertebral fractures usually happen in the elderly in a fall from a standing height or less while nonosteoporotic high-energy vertebral fractures occur in a relatively young patient in a high falling accident or a traffic collision. Finally, further studies with an animal model of osteoporosis need to be conducted to analyze the change in ASIC2 expression in the administration of antiresorptive drugs to treat osteoporosis.

## 5. Conclusions

Osteoblastogenesis was reduced in an acidic environment. ASIC2, ASIC3, and ASIC4 were most highly expressed in turn during osteoblastogenesis within acidic media. ASIC2 was the most abundantly expressed among the other ASICs in human bone cells in OVF compared with the control. ASIC2 may be crucial in the pathogenesis of osteoporosis and could serve as a therapeutic target for antiosteoporotic therapies.

## Figures and Tables

**Table 1:** Primer sequences for relative quantification of ASIC transcripts.

Gene	Forward primer	Reverse primer	Accession No.^*∗*^
ASIC1	CCCTGTTGTCTTGGTGAC	ATGGTGAGGTAGGATGTT	NM 020039
ASIC2	CGCCACTTCGAGGGAATCAG	GTAAACACGGAGGAGAAGTTGTG	NM 183377
ASIC3	GGTGTTCCGAGACAAGGTCC	GGTGGGAGGTCAGATTGGTG	NM 020322
ASIC4	GGTAGACATCCTCAACCGCA	GCGAGTATAGACCACAGAGAAGT	NM 182847
ALP	ACCATTCCCACGTCTTCACATTTG	AGACATTCTCTCGTTCACCGCC	NM 001127501
OPN	AGCCAGGACTCCATTGACTCGAAC	GTTTCAGCACTCTGGTCATCCAGC	NM 001040058
OC	ATGAGAGCCCTCACACTCCTC	GCCGTAGAAGCGCCGATAGGC	NM 199173
Na^+^/K^+^ ATPase	TGTCCAGAATTGCAGGTCTTTG	ACAACTGGTACTTGTTGGTGGA	NM 000701
GAPDH	GTCGGAGTCAACGGATTT	CAACAATATCCACTTTACCAGAG	NM 002046

*∗*Gene bank accession number of cDNA available on line at http://www.ncbi.nlm.nih.gov/;

ASIC: acid-sensing ion channel; ALP: alkaline phosphatase; OPN: osteopontin; OC: osteocalcin; GAPDH: glyceraldehyde-3-phosphate dehydrogenase

## Data Availability

The data used to support the findings of this study are available from the corresponding author upon request
